# ZnO-Based Amperometric Enzyme Biosensors

**DOI:** 10.3390/s100201216

**Published:** 2010-02-01

**Authors:** Zhiwei Zhao, Wei Lei, Xiaobing Zhang, Baoping Wang, Helong Jiang

**Affiliations:** 1 School of Electronic Science and Engineering, Southeast University, Nanjing 210096, China; E-Mails: lw@seu.edu.cn (W.L.); bell@seu.edu.cn (X.B.Z.); wbp@seu.edu.cn (B.P.W.); 2 State Key Laboratory of Lake Science and Environment, Nanjing Institute of Geography and Limnology, Chinese Academy of Sciences, Nanjing 210008, China

**Keywords:** ZnO, electrochemical, enzyme biosensors

## Abstract

Nanostructured ZnO with its unique properties could provide a suitable microenvironment for immobilization of enzymes while retaining their biological activity, and thus lead to an expanded use of this nanomaterial for the construction of electrochemical biosensors with enhanced analytical performance. ZnO-based enzyme electrochemical biosensors are summarized in several tables for an easy overview according to the target biosensing analyte (glucose, hydrogen peroxide, phenol and cholesterol), respectively. Moreover, recent developments in enzyme electrochemical biosensors based on ZnO nanomaterials are reviewed with an emphasis on the fabrications and features of ZnO, approaches for biosensor construction (e.g., modified electrodes and enzyme immobilization) and biosensor performances.

## 1. Introduction

A biosensor is an analytical device, which converts the modification of the physical or chemical properties of a biomatrix (e.g., enzyme, antibodies, receptors, organelles, microorganisms) into an electric or other kinds of signal whose amplitude depends on the concentration of defined analytes in the solution [1]. They are becoming essential in the field of healthcare, chemical and biological analysis, environmental monitoring, and food processing industries. According to the receptor type, biosensors can be classified as enzymatic biosensors, genosensors, immunosensors, *etc.* Biosensors can be also divided into several categories based on the transduction process, such as electrochemical, optical, piezoelectric, and thermal or calorimetric biosensors. Among these various kinds of biosensors, electrochemical biosensors are a class of the most widespread, numerous and successfully commercialized biomolecular electronic devices [1]. Particularly, enzyme-based electrochemical biosensors are attracting ever-increasing attention due to their potential applications in many areas [2,3].

One of the recent developments in the enzyme electrochemical biosensor field is based on combining the properties of biologically active substances with those of nanocrystalline materials. The nanostructure of these materials could provide high surface to volume ratios and high surface activity, and thus possess unique advantages over other conventional materials in terms of enzymatic immobilization and signal transduction. Nanomaterials could preserve enzyme activity due to the desirable microenvironment, and enhance the direct electron transfer between the enzyme’s active sites and the electrode.

Among nanomaterials, ZnO has attracted much attention due to its wide potential range of applications. As a wide band gap (3.37 eV) semiconductor, ZnO plays an important role in optics, optoelectronics, sensors, and actuators due to its semiconducting, piezoelectric, and pyroelectric properties [4,5]. Nanostructured ZnO not only possesses high surface area, good biocompatibility and chemical stability and is non-toxic, but it also shows biomimetic and high electron communication features [6–8], making it great for potential applications in biosensors. More importantly, as a biocompatible material, it has a high isoelectric point (IEP) of about 9.5. This makes nanostructured ZnO materials suitable for absorption of proteins with low IEPs, because the protein immobilization is primarily driven by electrostatic interaction. ZnO with various nanostructures prepared by different fabrication techniques, has been widely used for enzyme immobilization in recent years.

Recent advances in biocompatible nanomaterials and biotechnology open a promising field toward the development of enzyme-based biosensors. The present paper reviews the state-of-the-art of ZnO utilization for enzyme immobilization in electrochemical biosensors, key issues in ZnO synthesis methods and related features, biosensor construction (e.g., modified electrodes, enzyme immobilization) and biosensor performance. The content of this review is oriented toward covering biosensing of glucose, hydroperoxide, phenol, cholesterol, uric acid and urea, respectively.

## 2. ZnO-Based Enzyme Biosensing

### 2.1. Glucose

Glucose biosensor, as one of the most popular biosensors, has been intensively investigated due to its importance in clinics, environment and food industry. Glucose amperometric biosensor using glucose oxidase (GOD) as the enzyme is one of the most popular biosensors to be intensively investigated.

**Figure 1.**
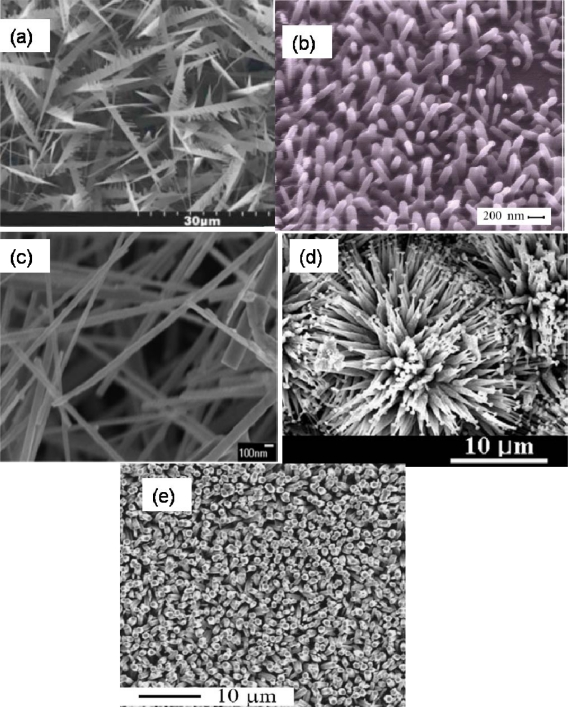
ZnO nanostructure materials with various shapes: (a) nanocombs made by vapor-phase-transport [13]; (b) nanowires obtained by vapor-liquid-solid [14]; (c) nanowires made by thermal evaporation [15]; (d) nanonails made by thermal evaporation [16]; (e) nanorods obtained by hydrothermal decomposition [17].

Application of ZnO nanostructure in the glucose biosensors just appeared in the last several years. Table 1 summarizes the state-of-the-art of ZnO utilization for enzyme immobilization in electrochemical biosensor platforms and their analytical performances. Physical adsorption is the mostly used method for enzyme immobilization. ZnO nanocomb, prepared by vapor-phase transport, was reported relatively early as a platform for glucose detection [13]. During the manufacturing process for ZnO nanocombs [Figure 1(a)], the temperature was controlled at 900 ^○^C. A mixture of ZnO and graphite powders was used as reaction raw material sources, and argon and oxygen were used as carrier gas and reaction gas, respectively. For enzyme immobilization, GOD was physically adsorbed onto the nanocomb modified Au electrode and covered by Nafion solution. The prepared biosensor had a diffusion-controlled electrochemical behavior and a fast response time, within 10 s. The value of K_M_ (Michaelis-Menten constant) was reported to be 2.19 mM. Using a similar technique, Weber *et al*. obtained ZnO nanowires with a typical length of 0.5–2 μm and a diameter of 40–120 nm [Figure 1(b)], which were grown from the substrate with an array of ZnO nanowires [14]. For the enzyme immobilization, physical adsorption was also adopted to immobilize GOD onto the electrode. Such a prepared biosensor had a wider linear range from 0.1 to 10 mM, compared to those of others [14].

ZnO nanowires can also be obtained using thermal evaporation, in which ZnS powders are thermally evaporated under controlled conditions with Au thin film as a catalyst layer [15]. GOD was immobilized onto ZnO nanowires [Figure 1(c)] via physical adsorption. K_M_ and sensitivity could be modulated over a wide range depending on the amount of ZnO/GOD loading on the electrode [15]. Other kinds of ZnO nanostructures, such as ZnO nanonails synthesized by thermal evaporation [Figure 1(d)] [16], are also proposed as a platform for enzyme immobilization. For ZnO nanonails, Zn powder was used as the source of Zn in the reaction, and oxygen was introduced into the system. The protocol used was similar to that as described in [13]. The constructed biosensor showed a high sensitivity of 24.6 μA/cm^2^·mM. It also exhibited a diffusion-controlled electrochemical behavior with a linear calibration range from 0.1 to 7.1 mM.

Among the various strategies used, a useful and simple way is to grow ZnO directly onto the electrode surface. Based on this approach, Wei *et al.* [17] fabricated ZnO nanorods directly on the standard Au electrode by hydrothermal decomposition. As shown in Figure 1(e), ZnO nanorods with a hexagonal cross section were uniform in size with a diameter of about 300 nm and a length of 4 μm. Enzyme immobilization was done by a cover of GOD solution on the surface of the electrode. The prepared biosensor presented a quite fast response (within 5 s) and a high sensitivity of 23 μA/cm^2^·mM. The K_M_ was as low as 2.9 mM.

Recently, Dai *et al.* reported tetragonal pyramid-shaped porous ZnO (TPSP-ZnO) nanostructures prepared by a wet chemical route [18]. A glassy carbon electrode (GCE) was modified by covering with a solution of a mixture of TPSP-ZnO and GOD. It showed a surface-controlled behavior. The biosensor had a wider linear response from 0.05 to 8.2 mM and also exhibited good stability and reproducibility. In addition, such a prepared biosensor did not suffer from interference by cooxidizable substances (e.g., ascorbic acid, uric acid and *p*-acetaminophen).

In addition to ZnO nanostructures as mentioned above, ZnO nanoclusters were also proposed as platforms for biosensor construction [19]. ZnO nanoclusters doped with Co (2%) were obtained by nanocluster-beam deposition [21,22]. Poly(ethyleneterephthalate) (PET) plate was used for enzyme immobilization instead of traditional standard electrode, which was modified firstly by Ti ions implantation and then covered by a thin Au layer. After that ZnO-based nanoclusters were directly grown on the modified PET plate. The mode of enzyme immobilization was also different from those previously reported. Cross-linking via glutaraldehyde was used for enzyme immobilization. The prepared biosensor had a response time under 10 s and the sensitivity was over 13 μA/ cm^2^·mM.

Recent advances in glucose biosensors involve introducing other nanomaterials with good properties to achieve better biosensor performance. Using this approach, a novel glucose biosensor was developed based on carbon-decorated ZnO nanowire arrays, which took advantage of the conductivity and chemical stability of carbon and the large specific surface area of ZnO nanowires [20]. ZnO nanowire arrays were grown directly on the electrode substrate, and the electron transfer resistance at the electrode was significantly reduced when carbon was introduced. Moreover, direct electron transfer occurred from GOD to carbon, and the ZnO nanowire array provided direct electron pathways for transferring electrons immediately from carbon to the electrode substrate. The developed biosensor had a fast response, within 5 s, and a high sensitivity of 35.3 μA/cm^2^·mM. A high affinity of GOD for glucose on the carbon-ZnO array (K_M_ = 1.54 mM) was found, and such a kind of biosensor showed good anti-interference properties towards ascorbic acid and dopamine.

### 2.2. H_2_O_2_

Over the past decades, the determination of hydrogen peroxide has been a very active study area because H_2_O_2_ plays an important role in the food industry, environmental monitoring and clinical diagnosis [23]. Electrochemical tracking of biological targets by way of enzyme-based H_2_O_2_ detection is of special interest. Comparing to other analytical techniques, such as spectrometry [24], titrimetry [25], and chemiluminescence [26], electrochemical enzyme biosensors have the advantages of high selectivity of the biological recognition elements and high sensitivity of electrochemical transduction process.

Table 2 summarizes H_2_O_2_ biosensors based on ZnO nanostructures using different approaches. A H_2_O_2_ biosensor was developed using waxberry-like ZnO microstructures consisting of nanorods (8–10 nm) as a platform [27]. These ZnO microstructures made by a wet chemical method possessed good biocompatibility without any damage to the secondary structure of the horseradish peroxidase (HRP) on the nano-ZnO/HRP electrode. Such ZnO nanomaterials with high surface areas could provide a platform for the reduction of H_2_O_2_ by contributing excess electroactive sites and thus providing enhanced electrocatalytic activity. The transport characteristics of the electrode were controlled by the diffusion process. This kind of biosensor had a much wider linear range, from 0.l5 to 15 mM, and a detection limit of 0.115 μM [27]. The modified electrode with carbon-decorated ZnO nanoarrays was also a good platform for H_2_O_2_ development. At the potential of −0.4 V, the biosensor showed a high sensitivity of 237.8 μA/cm^2^·mM and fast response of 4 s [20]. Interestingly, the catalytic activity of the enzymes could be improved by irradiating the surface of modified electrode with UV light and thus inducing a photovoltaic effect [28].

In recent years, nanostructured inorganic-organic hybrid materials have emerged for the fabrication of biosensors by entrapping enzymes, which are able to reserve the desirable properties of organic and inorganic materials to a large extent. Organic components (e.g., Nafion, chitosan) benefit the formation of defect-free inorganic membranes and make them less brittle, and the chemical and thermal stability of organic membranes can be improved by an inorganic phase [34–36]. Many efforts have been made recently in order to construct ZnO-based electrochemical biosensors with good performance. For example, a H_2_O_2_ biosensor with good stability was developed based on a nanoporous ZnO/chitosan inorganic-organic composite film as immobilization matrix [29]. HRP was entrapped in the ZnO/chitosan film. The sensor exhibited a sensitivity of 43.8 μA/cm^2^·mM, and could retain 80% of its initial current response after 40 days [29]. It was proposed that the numerous nanoscaled cavities on the surface of the microspheres are highly advantageous for the entrapment of enzymes by sequestering them in the cavities or binding on the surface of the microspheres. Based on this approach, Lu *et al.* synthesized the porous ZnO microspheres consisting of nanosheets using a wet chemical route [30]. Hemoglobin (Hb) was also entrapped in the composite film by thoroughly mixing a Hb solution, ZnO suspension and Nafion solution. In addition to the good reproducibility and long-term stability, the prepared biosensor had a high sensitivity of 137 μA/cm^2^·mM and a low K_M_ of 0.143 mM. Porous nanomaterials could provide a larger surface area for protein binding and decrease the diffusion distance for the substrate to access the immobilized enzyme, resulting in the higher sensitivity of the prepared biosensor [30].

Other nanomaterials, such as gold and multi-walled carbon nanotubes (MWCNTs), are also introduced into the hybrid inorganic-organic composites in order to take advantage of these inorganic nanomaterials. MWCNTs had been widely used to immobilize enzymes to realize direct electron transfer due to their excellent electronic properties [32]. After introducing MWCNTs into the composite system, the modified electrode could thus easily achieve the direct electron transfer between enzyme (e.g., Hb) and electrode. In addition, the presence of biocompatible Nafion in the biocomposite film not only makes the film uniform, but could also lead to an increased Hb activity [37]. Based on these ideas, a biosensor was prepared using the platform consisting of ZnO, MWCNTs and Nafion, which showed a very high sensitivity of 1310 μA/cm^2^·mM and a very low of K_M_ of 82.8 μM [32]. It’s known that gold can immobilize enzyme molecules while retaining good bioactivity and enhance the normal electron transfer kinetics of the enzyme electrode [23]. In the literature [31], gold nanoparticles were introduced into composites of ZnO and Nafion with HRP entrapped in the composites [31]. The biosensor had a fast response time under 5 s and a K_M_ of 1.76 mM. It possessed good reproducibility and showed good stability after one month. Another example based on composites was prepared by mixing of ZnO/chitosan solution and gold solution as immobilization platform; the as-prepared biosensor had a fast response to H_2_O_2_ within 4 s and excellent linear relationships from 0.19 μM to 1.73 mM [33].

It’s known that ZnO crystals with high IEP are suitable for the electrostatic adsorption of proteins with lower IEP. It is reasonable to immobilize enzyme via electrostatic adsorption and a good example could be found in the reference [23]. The positively-charged ZnO crystals and amine-derivatized chitosan could facilitate higher capability of assembling negatively charged nanogold through strong electrostatic adsorption and the covalent bonds between amine groups and gold [23]. Biocompatible nanogold could further allow HRP to be immobilized with well-retained bioactivity in addition to increased loading amount. The so prepared biosensor can achieve sensitive electrochemical response to H_2_O_2_ at a potential of −0.2 V.

### 2.3. Phenol

Phenolic compounds often exist in the wastewaters of many industries, giving rise to environmental problems as many of them are very toxic. The sensitive monitoring of phenolic compounds is thus an important subject for environmental monitoring and a great amount of effort has been devoted to the development of simple and effective analytical methods for the determination of phenolic compounds. Electrochemical detection is the most promoted method due to its low cost for realization and the potential for miniaturization and automation. Tyrosinase is an enzyme with a low IEP of 4.6 [38]. It has good catalyzing selectivity for detection of phenolic compounds and is generally immobilized on electrode surfaces to set up electrochemical biosensors [39]. Generally, the immobilization of tyrosinase is a key step to fabricate a phenol biosensor. The biomolecules of tyrosinase with low IEP could be adsorbed onto ZnO nanostructures with high IEP. Phenol biosensors have thus been developed based on the electrostatic attraction between electropositive ZnO nanostructures and tyrosinase.

The summary of phenol biosensors based on ZnO nanostructures is shown in Table 3. The working potential was mostly fixed at −0.2 V. A reagentless phenol biosensor was prepared by immobilizing tyrosinase on ZnO nanorods through electrostatic attraction and then covered by Nafion, in which ZnO nanorods were fabricated by vapor-phase transport [39]. The electrode reaction was a diffusion-controlled quasi-reversible process. Tyrosinase was adsorbed on the ZnO nanorods and still retained its bioactivity. The biosensor had a fast response of under 5 s and the linear range of concentration spanned from 0.02 to 0.18 mM. A similar strategy for tyrosinase immobilization onto ZnO nanorods by a hydrothermal reaction was reported by Gu *et al.* [40]. The prepared biosensor had a similar response time below 5 s. There were two different ranges for the sensitivity and linear response and the K_M_ value was as low as 0.17 μM.

ZnO matrix made by a sol-gel procedure was developed as a platform for tyrosinase immobilization [42]. The porous and positively charged ZnO sol-gel matrix provided a moderate microenvironment for tyrosinase to retain its bioactivity. The so prepared biosensor had a sensitivity of 168 μA/mM, and the linear range covered from 0.15 to 40 μM [42]. Another matrix of ZnO/chitosan was also developed by the same research group for tyrosinase immobilization by dispersion of ZnO nanoparticles into the chitosan solution [41]. The matrix could provide a favored microenvironment in terms of its favorable isoelectric point for tyrosinase loading and the immobilized tyrosinase could retain its bioactivity to a large extent. The biosensor using ZnO/chitosan matrix had a better performance than that using the ZnO sol-gel matrix. K_M_ was calculated to be 23 μM and the detection limit was lower than 0.05 μM [41]. Recent advances in phenol biosensors include the use of modern processes widely used in semiconductor industry, such as photolithography for designed patterns. A new tyrosinase biosensor was reported based on the covalent immobilization of tyrosinase by glutaraldehyde on biofunctional ZnO nanorod microarrays [43]. The as-prepared biosensor had a sensitivity of 287 μA/cm^2^·mM and a detection limit of 0.25 μM. The linearity spanned a wide range from 1–150 μM.

### 2.4. Cholesterol

Cholesterol and its fatty acid esters are one of the main constituents in human beings as they are the components of nerve and brain cells [44], and are the precursors for other biological materials, such as bile acid and steroid hormones [45]. It is therefore desirable to develop a reliable and sensitive biosensor which can allow for a convenient and rapid determination of cholesterol [46].

Table 4 summarizes the developed cholesterol biosensors using ZnO as platform. Obviously, physical adsorption was generally accepted for cholesterol oxidase (ChOx) immobilization. It is interesting to use porous ZnO thin films as platform for enzyme immobilization [47]. Different from traditional ZnO thin films obtained by RF magnetron sputtering, the ZnO thin film was grown under high pressure (50 mTorr) so as to create native defects and therefore a porous film was formed. The prepared biosensor had a K_M_ of 2.1 mM and a much wider linear range from 0.65 to 10.34 mM. Cholesterol biosensors could be also constructed using ZnO nanoparticles made by a wet chemical route as platform; they had a high and reproducible sensitivity of 23.7 μA/cm^2^·mM and a detection limit of 0.37 nA [46].

Recently, an ultra-sensitive cholesterol biosensor was developed using flowerlike ZnO structures. These flowerlike ZnO nanostructures was synthesized by a chemical route [48]. ChOx was immobilized onto the surface of the modified electrode by physical adsorption, followed by covering of the modified electrode with a Nafion solution. The fabricated sensor exhibited a very high and reproducible sensitivity of 61.7 μA/cm^2^·mM with a detection limit of 0.012 μM. It also had a K_M_ of 2.57 mM and a fast response time of 5 s [48].

For cholesterol biosensors, an inorganic-organic nanocomposite thin film which consisted of ZnO nanoparticles and chitosan was also proposed to modify electrodes [49]. The resulting ChOx/ZnO-chitosan/ITO bioelectrode exhibited a very low K_M_ value of 0.22 mM, suggesting the high affinity of the enzyme on the nanocomposite towards cholesterol.

### 2.5. Others

In addition to the above ZnO-based biosensors for substance detection (e.g., glucose, H_2_O_2_, phenol and cholesterol), other biosensors have also been developed to detect substances including uric acid and urea. The research group of Zhang *et al.* reported relatively early a uric acid biosensor based on ZnO nanorods formed by thermal evaporation [50]. Uricase, to catalyze the uric acid with a low IEP of 4.3, was immobilized on ZnO nanorods by electrostatic attraction. In addition to a low K_M_ of 0.24 mM, the prepared biosensor had a linear range from 5 μM to 1 mM and a detection limit of 2 μM according to differential pulse voltammetry (DPV) response investigation. Besides, it had a good thermal stability (10–85 ^○^C), indicating that such biosensor could withstand elevations in temperature frequently in excess of those that normally denature the native enzyme [51,52]. A recent development in the field of ZnO-based uric acid biosensors is a highly sensitive and stable uric acid biosensor based on a multilayer structure. Using ZnO nanoparticles and MWCNTs, a multilayer structure was realized firstly with negatively charged MWCNTs cast on pyrolytic wafers, followed by the decoration of ZnO nanoparticles [53]. Uricase was immobilized onto ZnO nanoparticles also by electrostatic attraction, and finally a PDDA layer was coated on the uricase surface. The as-prepared biosensor had a wide linear response range from 1 mM to 5 M and a high sensitivity of 393 μA/cm^2^·mM. Its long-term stability can over 160 days.

Up to now, not much has been published on ZnO-based urea biosensors. Urea [(NH_2_)_2_CO] is basically an organic compound of carbon, nitrogen, oxygen and hydrogen. Most organisms use thus route to deal with the excretion of nitrogen originating from protein and amino acid catabolism. Recently, Ansari *et al.* reported a urea biosensor constructed from ZnO nanostructures [54]. ZnO was synthesized by a chemical route and urease was covalently attached on the ZnO nanostructure. The sensor had a fast response time of 6 s and a linear range of 10–50 mM.

## 3. Discussion

It is known that nanomaterials play an important role in biosensor construction. Besides ZnO materials, carbon nanotubes (CNTs) and gold nanoparticles are another two materials popularly used for biosensor construction. It’s known that CNTs are one-dimensional materials with unique properties such as good electrical conductivity, strong adsorptive ability and excellent bioconsistency. The applications of CNTs in the biosensors have shown that CNTs have an electrocatalytic effect and fast electron-transfer rates between the electroactive species and the electrode. For gold, they could provide a stable immobilization for biomolecules retaining their bioactivity. Electron transfer between redox proteins and electron surfaces is facilitated by many factors, such as the high surface-to-volume ratio, high surface energy, decreased proteins-metal particles distance and the functioning as electron-conducting pathways.

CNTs were synthesized originally by arc discharge and later laser ablation was developed. Recently, chemical vapor deposition has become a promising technique for CNT growth, which can produce large amount CNTs with good properties. However, before CNT growth, a catalyst layer or catalyst particles (e.g., Ni, Co, Fe) normally have to be induced, thus making this process more complex. Besides, impurities (e.g., amorphous carbon, graphite nanoparticles, metal catalysts and catalyst support materials) contained in the produced CNTs handicap the development of further applications. Therefore, the post-treatment for the CNT products including purification methods are needed. Gold nanoparticles are normally obtained by a chemical route and electrodeposition. Compared to CNTs and gold nanoparticles, there are more traditional methods currently used for ZnO synthesis. In addition to the wet chemical route also for gold growth and vapor phase transportation similar to that for CNT growth, other methods including hydrothermal decomposition, sol-gel, thermal evaporation and thin film deposition techniques are also developed (see Tables 1–4).

The numerous choices for ZnO fabrication and also their different growth parameters have led to a rather rich ZnO nanoworld consisting of nanostructures with different shapes (e.g., nanocombs, nanowires, nanobelts, nanorods, nanosheets, nanonails, nanoparticles, nanoneedles, nanoflowers, *etc.*). This variety of ZnO shapes results in formation of different structures and thus various properties are exhibited, which might further influence the microenvironments after an enzyme is immobilized. These various ZnO nanostructures in different shapes are also favorable for surface functioning if needed. Unlike ZnO, CNTs and gold nanostructures normally only have a limited range of structures available for biosensor construction. For example, gold nanoparticles normally must be used for the construction of biosensors.

To construct a biosensor with promising applications, modification of the electrode in an effective way should be carefully considered. The immobilization of enzyme onto the electrodes should be considered as a key step due to the important roles of the amount and bioactivity of immobilized enzyme on the performance of biosensors. For ZnO-based biosensors (e.g., glucose and cholesterol biosensors), enzyme immobilization by physical adsorption is normally used due to its simplicity. The high IEP of ZnO makes it a good matrix to immobilize acidic proteins by electrostatic interactions with high binding stability and insignificant protein denaturization. For example, tyrosinase with low IEP of 4.6 is immobilized onto ZnO by electrostatic attraction due to the negative charge of tyrosinase and the positive charge on ZnO under working buffer pH. For CNTs- and gold- based biosensors, electrostatic attraction is normally used where multilayered systems are proposed via layer by layer technology. The bioelectrocatalytic response was directly correlated to the number of deposited bilayers, and some of the biosensors prepared this way exhibited high K_M_ values [55,56].

Generally, both CNTs and gold have better conductivity than ZnO, but the good performance of many ZnO-based biosensors has been presented (see Tables 1–4). For example, they could show very high sensitivity, lower K_M_, and fast response times, even under 5 s. The high performance of the sensors could be ascribed to the use of ZnO with various nanostructures related to high specific surface area and also its high IEP, which allows the effective immobilization of a larger amount of the enzyme.

For practical applications, one of the challenges currently faced is that the construction of a low cost biosensor is still essential when considering the commercial devices. Obviously, gold-based amperometric enzyme biosensors have to overcome this for practical applications.

## 4. Conclusions

This review presented the development of several ZnO-based electrochemical biosensors (e.g., glucose, hydrogen peroxide, phenol, cholesterol, *etc.*) with an emphasis on ZnO fabrication techniques, enzyme immobilization methods and biosensor performance. The comparative features of the biosensors have been summarized in several tables for an easier overview. The effects of the unique properties of various ZnO nanomaterials on the immobilization of enzymes that retain their biological activity make them a powerful tool to modify electrodes for the construction of sensitive biosensors with advantageous features, which may find applications in many fields of interest.

## Figures and Tables

**Table 1.** ZnO-based electrochemical biosensors for glucose detection (GOD as enzyme) (GCE: glass carbon electrode; Gold/Ti/PET: Gold/Ti/poly (ethyleneterephthalate); “/” in the table means the corresponding content is not provided.)

Ref.	[13]	[14]	[15]	[16]	[17]	[18]	[19]	[20]
Electrode	Gold	/	gold	gold	gold	GCE	Gold/Ti/PET	Ti
Fabrication of ZnO	Vapor-phase transport	Vapor-liquid-solid	Thermal evaporation	Thermal evaporation	hydrothermal	Wet chemical route	Nanocluster-beam	Wet chemical route
ZnO structure	nanocombs	nanowires	nanowires	nanonails	nanorods	nanoparticles	nanoparticles	carbon-decorated nanowires
Immobilization mode	Physical adsorption	Physical adsorption	Physical adsorption	Physical adsorption	Physical adsorption	Physical adsorption	Cross-linking	Physical adsorption
Working potential (V)	+0.8	+0.8	+0.8	+0.8	+0.8	−0.5	+0.8	−0.45
Response time (s)	<10	/	<10	<10	<5	/	<8	<5
K_M_ (mM)	2.19	/	2.1–11.8	15	2.9	/	21	1.54
Sensitivity (μA/ cm^2^·mM)	15.33	/	26–0.8	24.6	23.1	/	13.3	35.3
Linear response range (mM)	0.02–4.5	0.1–10	/	0.1–7.1	0.01–3.45	0.05–8.2	0–4	0.01–1.6
detection Limit (μM)	20	/	0.7	5	10	10	20	1

**Table 2.** ZnO-based electrochemical biosensors for hydrogen peroxide detection (HRP: horseradish peroxidase; Hb: hemoglobin; MP: microperoxidase; “/” in the table means the corresponding content is not provided.)

Ref.	[27]	[20]	[28]	[29]	[30]	[31]	[32]	[23]	[33]
Enzyme/Electrode	HRP GCE	HRP Ti	MP graphite	HRP GCE	Hb GCE	HRP GCE	Hb GCE	HRP GCE	Hb GCE
Fabrication of ZnO	Wet chemical	Wet chemical	commercial	commercial	Wet chemical	Wet chemical	Hydro-thermal	Hydro-thermal	Wet chemical
ZnO structure	nanorods	nanowires	nanoparticles	nanoporous	nanosheet	flowerlike	Thin film	crystals	nanoparticles
Immobilization mode	Chemical adsorption	Physical adsorption	Entrapped	Entrapped	Entrapped	Entrapped	Entrapped	Electrostatic attraction	Entrapped
Working potential (V)	/	−0.4	/	/	−0.675	−0.3	−0.39	−0.2	−0.28
Response time (s)	/	4	1.5	<10	/	<5	/	<10	<4
K_M_ (mM)	/	/	/	/	0.143	1.76	0.0828	/	0.075
Sensitivity (μA/ cm^2^·mM)	/	237.8	0.041 μA/ mM	43.8	137	/	1310	369	/
Linear response range (μM)	150–15000	/	0.1–800	5–2000	1–410	10–1100	0.2–12	1.5–450	0.19–1730
Detection Limit (μM)	0.115	0.2	0.03	2.5	10	9	0.084	0.7	0.097

**Table 3.** ZnO-based electrochemical biosensors for phenol detection (tyrosinase as enzyme) (“/” in the table means the corresponding data is not provided.).

Ref.	[39]	[40]	[41]	[42]	[43]
Electrode	GCE	Treated gold sphere	GCE	GCE	Nanocrystalline diamond
Fabrication of ZnO	Vapor-phase transport	Hydrothermal	Hydrothermal	Sol-gel	Chemical route
ZnO structure	nanorods	nanorods	nanoparticles	ZnO sol-gel solution	ZnO nanorod microarray
Immobilization mode	Electrostatic attraction	Electrostatic attraction	Electrostatic attraction	Electrostatic attraction	Covalent binding
Working potential (V)	−0.2	−0.2	−0.2	−0.2	−0.15
Response time (s)	<5	<5	<10	<15	/
K_M_ (mM)	0.24	0.17 × 10^−3^	23 × 10^−3^	/	/
Sensitivity (μA/mM)	0.83	40 (<20μM) 103 (>20 μM)	182	168	287 (μA/cm^2^·mM)
Linear response range (μM)	20–180	two ranges	0.15–65	0.15–40	1–150
Detection Limit (μM)	15.57	0.623	0.05	0.08	0.25

**Table 4.** ZnO-based electrochemical biosensors for cholesterol detection (cholesterol oxidase as enzyme) (ITO: Indium tin oxide; “/” in the table means the corresponding content is not provided.).

Ref.	[47]	[46]	[48]	[49]
Electrode	Gold-coated ITO	Gold	Gold	ITO
Fabrication of ZnO	Magnetron sputtering	Chemical route	Chemical route	Chemical route
ZnO structure	Porous thin film	nanoparticles	Flowerlike ZnO (nanorods)	nanoparticles
Immobilization mode	Physical adsorption	Physical adsorption	Physical adsorption	Physical adsorption
Response time (s)	<15	<5	<5	15
K_M_ (mM)	2.1	4.7	2.57	0.22
Sensitivity (μA/cm^2^·mM)	/	23.7	61.7	3.6
Linear response range (mM)	0.65−10.34	1–500 × 10^−3^	1–15 × 10^−3^	0.13−7.77
detection Limit (μM)	/	0.37 × 10^−3^	0.012	130
